# Genomic characterization of a local epidemic *Pseudomonas aeruginosa* reveals specific features of the widespread clone ST395

**DOI:** 10.1099/mgen.0.000129

**Published:** 2017-06-08

**Authors:** Marie Petitjean, Daniel Martak, Alicia Silvant, Xavier Bertrand, Benoit Valot, Didier Hocquet

**Affiliations:** ^1^​ Hygiène Hospitalière, Centre Hospitalier Régional Universitaire de Besançon, Besançon, France; ^2^​ UMR CNRS 6249 Chrono-Environnement, Université de Bourgogne Franche-Comté, Besançon, France; ^3^​ Centre de Ressources Biologiques - Filière Microbiologique, Centre Hospitalier Universitaire de Besançon, 3 Boulevard Fleming, Besançon, France

**Keywords:** *Pseudomonas aeruginosa*, multidrug resistance, copper, outbreak, high-risk clone

## Abstract

*Pseudomonas aeruginosa* is a ubiquitous opportunistic pathogen with several clones being frequently associated with outbreaks in hospital settings. ST395 is among these so-called ‘international’ clones. We aimed here to define the biological features that could have helped the implantation and spread of the clone ST395 in hospital settings. The complete genome of a multidrug resistant index isolate (DHS01) of a large hospital outbreak was analysed. We identified DHS01-specific genetic elements, among which were identified those shared with a panel of six independent ST395 isolates responsible for outbreaks in other hospitals. DHS01 has the fifth largest chromosome of the species (7.1 Mbp), with most of its 1555 accessory genes borne by either genomic islands (GIs, *n*=48) or integrative and conjugative elements (ICEs, *n*=5). DHS01 is multidrug resistant mostly due to chromosomal mutations. It displayed signatures of adaptation to chronic infection in part due to the loss of a 131 kbp chromosomal fragment. Four GIs were specific to the clone ST395 and contained genes involved in metabolism (GI-4), in virulence (GI-6) and in resistance to copper (GI-7). GI-7 harboured an array of six copper transporters and was shared with non-pathogenic *Pseudomonas* sp. retrieved from copper-contaminated environments. Copper resistance was confirmed phenotypically in all other ST395 isolates and possibly accounted for the spreading capability of the clone in hospital outbreaks, where water networks have been incriminated. This suggests that genes transferred from copper-polluted environments may have favoured the implantation and spread of the international clone *P. aeruginosa* ST395 in hospital settings.

## Abbreviations

CRISPR-Cas, clustered regularly interspaced short palindromic repeats-CRISPR associated; GI, genomic island; ICE, integrative and conjugative element; NCBI, National Center for Biotechnology Information; ncRNA, non-coding RNA; QRDR, quinolone-resistance-determining region; ST, sequence type.

## Data Summary

1. This whole-genome sequencing project has been deposited at GenBank/EMBL/DDBJ; accession number: CP013993.1 (url – https://www.ncbi.nlm.nih.gov/nuccore/CP013993.1).

2. The collections of ST395 and non-ST395 isolates have been deposited at GenBank/EMBL/DDBJ; BioProjects: PRJNA379554 (url – https://www.ncbi.nlm.nih.gov/bioproject/?term=PRJNA379554) and PRJNA380885 (url – https://www.ncbi.nlm.nih.gov/bioproject/?term=PRJNA380885) (Table S2, available in the online Supplementary Material).

3. The 82 *Pseudomonas aeruginosa* isolates for which complete genomes were available from the National Center for Biotechnology Information at the time of the study (June 2017) were included in the study.

## Impact Statement

The opportunist pathogen *Pseudomonas aeruginosa* has a panmictic population structure, with a few international clones being frequently associated with outbreaks in hospitals worldwide. The biological features that favour the implantation and spread of these clones in hospital settings are largely unknown. ST395, one of these clones, has been retrieved from polluted environments and found to be responsible for outbreaks in hospitals. The genomic analysis of a collection of independent ST395 isolates identified four specific genomic islands (GIs) that contained genes involved in metabolism, virulence and the resistance to copper. An array of six copper transporters was borne on a single GI that was shared with non-pathogenic *Pseudomonas* sp. found in copper-contaminated environments. The systematic resistance to copper of ST395 isolates possibly accounted for their spreading capability in hospital outbreaks, where water networks have been incriminated. This observation could help implement infection control measures to tackle the proliferation of copper-resistant pathogenic bacteria in water networks.

## Introduction

*Pseudomonas aeruginosa* is an opportunistic human pathogen responsible for outbreaks in hospital settings [1]. This bacterial pathogen infects injured, burned, immunodeficient, immunocompromised and cystic fibrosis patients [2]. Despite the non-clonal epidemic population structure of *P. aeruginosa*, several multi-locus sequence types (STs) spread worldwide and are frequently associated with epidemics where multidrug resistance confounds treatment. ST111, ST175, ST235 and ST395 are among these so-called ‘international’ or ‘high-risk’ clones [3]. The biological features that make these clones more epidemic than other STs are largely unknown.

We focused here on the high-risk clone ST395, for which spread has been documented within hospitals in France and in the UK [4, 5]. From 1997 to 2008, a strain of *P. aeruginosa* ST395 has infected or colonized more than 300 patients in the University Hospital of Besançon (Besançon, France) [6]. Similarly, the clone ST395 was responsible for an outbreak in a UK hospital. The authors suggested that *P. aeruginosa* ST395 rapidly colonized the plumbing system of a new hospital, before its commissioning, and was transmitted to burn patients with the water [5]. Besides this, the clone ST395 has also been retrieved in a plant from a metal-contaminated estuary, and harboured resistance determinants to mercury, arsenic and copper, together with antibiotic-resistance genes [7].

*P.* *aeruginosa* strains adapt to environments through the gain or loss of blocks of genes [i.e. horizontal gene transfers of genomic islands (GIs) or integrative and conjugative elements (ICEs)] and modifications at the nucleotide level. Acquired genes originate from either clinical or environmental species [8].

The virulence determinants are mostly part of the core genome of the species, accounting for the ability of *P. aeruginosa* to inhabit a wide range of environments, including patients [9]. Moreover, the co-selection of antibiotic-resistance genes with metal-resistance genes can promote the ability of strains adapted to toxic environments to thrive in hospital settings, where the antibiotic consumption is high [10].

We aimed here to define the features that could have helped the implantation and spread of the clone *P. aeruginosa* ST395 in hospital settings. We first thoroughly analysed the complete genome of the multidrug-resistant index isolate of an outbreak in France (isolate DHS01), and identified genetic elements specific to this isolate, paying particular attention to the resistome, the virulome and mobile genetic elements. Among these specific elements, we further identified those shared with a panel of independent ST395 isolates responsible for outbreaks in other hospitals.

## Methods

### Genome sequencing of the index isolate

The isolate DHS01, the first isolate of the epidemic, was isolated in May 1997 from the nose of a 31-year-old male patient in the surgical intensive care unit in the University Hospital of Besançon (Eastern France). In our hospital, *P. aeruginosa* is searched for weekly in patients hospitalized in the intensive care unit. Microbiological records revealed that the patient was already colonized by *P. aeruginosa* 25 days before isolation of DHS01. DHS01 was identified at the species level by matrix-assisted laser desorption ionization-time of flight mass spectrometry (MALDI-TOF MS) with a Microflex LT (Bruker Daltonik), according to the manufacturer's procedures. Bacterial DNA was isolated from an overnight culture on Mueller-Hinton (MH) agar using a Genomic-tip kit (Qiagen) and further sequenced with Single Molecule Real-Time technology (Pacific Biosciences) [11]. The reads were assembled using Hgap3 into a circular complete genome [12]. The genome was annotated using the National Center for Biotechnology Information (NCBI) Prokaryotic Genome Annotation Pipeline (pgat) [13]. The genes were sorted as core or accessory genes of the species *P. aeruginosa* by comparing to previous pangenome analysis [14].

### Dataset

We compared the DHS01 genome against two datasets. Dataset 1 included 82 *P* *. aeruginosa* isolates which complete genomes were available on NCBI at the time of the study (June 2017; Table S1). Dataset 2 included the raw reads of 6 ST395 isolates of *P. aeruginosa* and 10 non-ST395 isolates of *P. aeruginosa* isolated during outbreaks in French or UK hospitals (Table S2) sequenced with the Illumina NextSeq technology with a mean depth of 200X coverage per genome. The absence of close relatedness between the ST395 isolates (*n*=7) was determined by pulsed-field gel electrophoresis, as described elsewhere (Fig. S1) [15].

### Phylogenetic analysis

We determined the ST of DHS01 with the Center for Genomic Epidemiology website (2017; https://cge.cbs.dtu.dk//services/MLST/). Then, the DHS01 genome was compared with the genomes of dataset 1 (Table S1) with MUMmer [16]. The length of the core genome alignment was 1770 651 bp, representing 1973 genes with 118 840 variable positions. The phylogenetic tree was determined using PhyML software [17] with the following parameters: GTR (general time reversible) substitution model with gamma-distributed rate variation across sites and a proportion of invariable sites.

### Mobile genetic elements

GIs and CRISPR-Cas (clustered regularly interspaced short palindromic repeats-CRISPR associated) systems were located with IslandViewer and CRISPRfinder, respectively [18, 19]. ICEs were retrieved from gene annotation (i.e. containing an integrase-encoding gene at the 5′-end, genes encoding the full conjugation machinery *traG* and *trbBCDEFGHI*, and a relaxase). Free circular forms of the ICEs were detected in the DNA extracted from SOS-induced bacteria by nested PCR using primers that overlapped the *attI* sequence (Table S3). The SOS response was induced by mitomycin, as previously described [20]. To identify ST395-specific GIs and ICEs, we mapped the Illumina reads from a collection of ST395 and non-ST395 isolates (Table S1) on the genome of DHS01. The presence of GIs and ICEs was determined with featureCount.

### Identification of resistance and virulence determinants

The susceptibility to the major antipseudomonal agents (ticarcillin, piperacillin, cefepime, ceftazidime, aztreonam, imipenem, amikacin, gentamicin, tobramycin and ciprofloxacin) was tested using the European Committee on Antimicrobial Susceptibility Testing recommendations with the reference agar dilution method, with the reference strain PAO1 as a control [21]. Resistance genes were searched for with ResFinder [22]. We searched for chromosomal mutations, in comparison with the genome of PAO1, responsible for resistance to antibiotics in the gene *oprD*, those encoding the main efflux systems (MexAB–OprM, MexXY, MexCD–OprJ, MexEF–OprN) and their regulators (MexR, MexZ, ArmZ, NfxB, NalC, NalD, ParS, ParR, MexS and MexT), the quinolone-resistance-determining regions (QRDRs; in *gyrA*, *gyrB*, *parC*, *parE*), the gene encoding the AmpC cephalosporinase (*ampC*) and its regulators (*dacB*, *ampR*, *ampD*, *ampDh2*, *ampDh3, ampG, ampO* and *ampP*), the regulators of polymyxin resistance (*colR*, *colS*, *cprR*, *cprS*, *pmrA* and *pmrB*) and the *phoP–phoQ* genes associated with colistin resistance. Virulence genes were searched for using the virulence factor database (vfdb) [23]. Mutations in resistance and virulence genes were detected using blast [24]. We first determined the serotype of DHS01 *in silico* using the PAst (*P. aeruginosa* serotyper) online tool from the Center for Genomic Epidemiology (2015; https://cge.cbs.dtu.dk/services/PAst-1.0/) and confirmed the result with specific antiserum (BioRad).

### Survival in copper solution

The bacterial survival in 7.5 mM CuSO_4_ was determined by the inoculation of cation-calibrated MH broth (Becton-Dickinson) with 10^7^ c.f.u. stationary-phase cells ml^−1^ and incubation for 48 h at 37 °C under shaking conditions (150 r.p.m.). The initial and final concentrations of living bacteria were determined by counting on MH agar plates. Experiments were performed at least three times independently.

## Results and Discussion

### Genome assembly

**Fig. 1.**
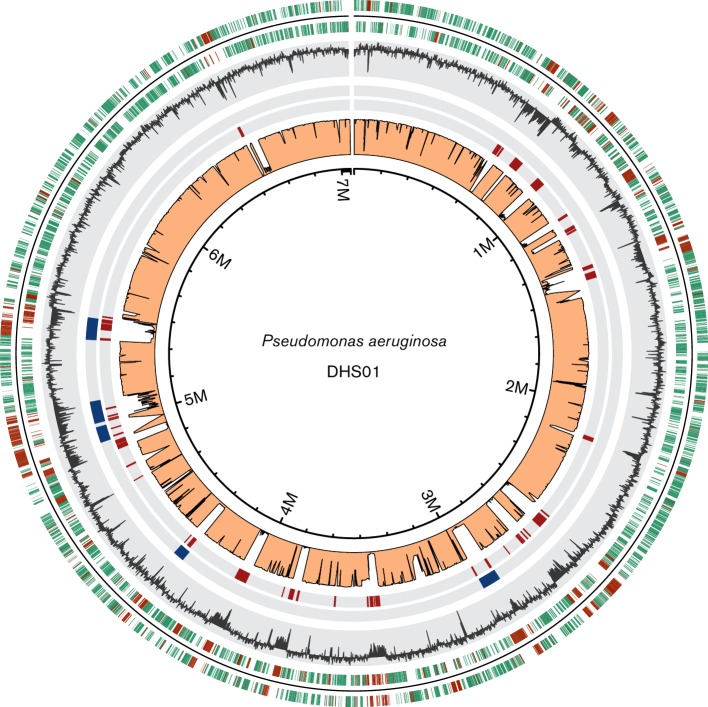
Circle plot of the complete genome of the epidemic *P. aeruginosa* DHS01 (ST395). The first and second outer circles indicate the coding DNA sequences on the plus and the minus strand, respectively. Genes in green are part of the core genome of the species and genes in brown are accessory genes. The third circle displays the G+C content (deviation from the mean) of the DHS01 genome. The two next circles show the location of GIs (in red) and ICEs (in blue). The pink innermost circle displays the numbers of genomes (of the 82 genomes of the dataset 1) containing this sequence. We drew the circle plot with R software.

The sequencing of the DHS01 genome generated 98 987 passed filter reads, with a mean read length of 12 kb and a mean coverage of 134×. The quality of the data allowed the assembly of the DHS01 genome in a complete circular chromosome of 7 055 752 bp (Fig. 1). Plasmids were absent from the genome. pgat annotation predicted 6524 coding genes, 67 tRNAs, 5 non-coding RNAs (ncRNAs) and 12 rRNAs (4 5S, 4 16S and 4 23S). The G+C content of the DHS01 genome (65.8 mol%) was low compared with that usually found in the species (~66.6 mol%). The genes acquired by horizontal transfer have generally a lower G+C content than that of the core genome. The size of the core genome of *P. aeruginosa* has been evaluated at 5234 genes [14]. By clustering with CD-HIT (cluster database at high identity with tolerance), we found that 1555 genes formed the accessory genome of DHS01. We found that 866 out of 1555 accessory genes (56 %) were borne by either GIs or ICEs, confirming that these mobile genetic elements were a major source of accessory genome and genetic diversity [8, 25]. The distribution of core and accessory genes is indicated in the Fig. 1.

### Phylogenetic analysis

DHS01 belonged to ST395 and was predicted to be of serotype O6. DHS01 has the fifth largest chromosome when compared with those of other isolates of the species, suggesting the recent integration of genetic material in its genome. The three largest chromosomes of the species belonged to ST111 isolates, the fourth largest belonged to a ST357 isolate and the sixth largest belonged to a ST235 isolate. Interestingly, all these STs are responsible for large outbreaks in hospital settings. This observation was in line with recent data showing that the genomes of ST111, ST395 and ST235 isolates were of 7.05, 7.00 and 6.81 Mbp, respectively, while the mean genome of the species was 6.72 Mbp in size [26].

**Fig. 2.**
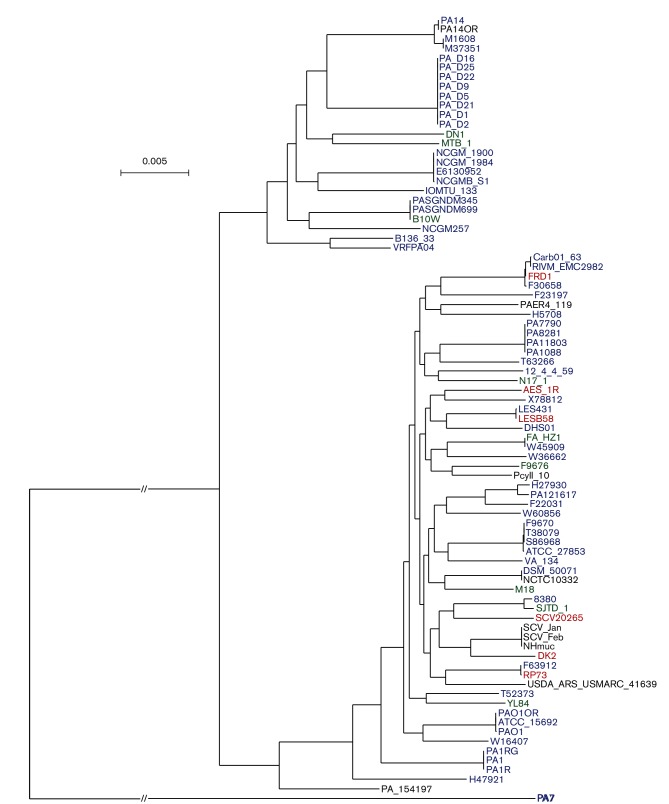
Phylogenetic tree of 83 isolates of *P. aeruginosa* with complete genomes, including DHS01. The phylogenetic tree is well supported, with the majority of branch support values higher than 98. The isolates cultured from cystic fibrosis patients are coloured red, non-cystic fibrosis clinical isolates are coloured blue, isolates of environmental origin are written in green and isolates of other origin are written in black. Bar, 0.005 substitution per site.

The phylogenetic analysis of 83 *P* *. aeruginosa* isolates (DHS01 and those of dataset 1) confirmed the split into two clusters containing either reference strains PAO1 or PA14 (Fig. 2) [27]. When compared to the PAO1 genome, the DHS01 genome expectedly displayed the major chromosomal inversion between two copies of ribosomal RNA reported in PA14 and all the genomes tested by Lee *et al.* [27]. DHS01 had 5833 and 5837 SNPs with its closest isolates LESB58 and LES431, respectively, that belonged to the highly virulent epidemic strain (LES, Liverpool Epidemic Strain) [28].

### DHS01 has undergone a 131 kb chromosomal deletion

DHS01 was the first isolate of a 11 year outbreak in the University Hospital of Besançon and was then considered as an ancestor from which the late isolates derived [6]. However, when compared with the genome of DHS29, a late isolate of the outbreak, the DHS01 genome had undergone a 131 kb chromosomal deletion [29]. This segment was intact in the genomes of five of six independent ST395 isolates (dataset 2, Table S2). The 131 kb gene block included the genomic regions PA1246–PA1292 and PA1381–PA1450 in the reference strain PAO1, and encompassed the virulence genes encoding the alkaline protease AprA (that degrades components of the host immune system) and its secretion system AprDEFI. The deletion also included the genes encoding the biofilm dispersion regulator BdlA, the quorum-sensing master regulator LasR, the LasI enzyme and the quorum sensing repressor RsaL, and the flagellar operon region including *fliKLMNOPQR* and *flhB* [30, 31]. Based on published phenotypic observations, one can hypothesize that the loss of these genes certainly affects the biofilm formation, the virulence, the adherence and the motility of DHS01 [32, 33].

### DHS01 virulence factors

We compared the virulome of DHS01 with that of the reference strain PAO1 using the virulence factor database (vfdb). The 347 virulence genes found in DHS01 were also retrieved from PAO1. Unexpectedly, 36 virulence genes present in PAO1 and part of the core genome of the species were missing from the DHS01 genome [14]. The 131 kb chromosomal deletion described above included 18 of these genes. Other genes missing from the DHS01 chromosome were implicated in bacterial adherence and biofilm formation. Hence, eight virulence genes contiguous in PAO1 (*flgK*, *flgL*, *fgtA*, *fliC*, *flaG*, *fliD*, *fliS* and *fleP*) and forming a block of 14 846 nt were missing from DHS01, probably as the result of a deletion ‘*en bloc*’. Besides this, the genes encoding the chaperonins CupE3 and CupC1 displayed 6 nt and 5 nt deletions, respectively, when compared with PAO1. Genes implicated in the synthesis of type IV pili were missing (*pilA*, only 17 % of the sequence was retrieved) or mutated (*pilB* and *pilC*). The deletion of the type IV pili-related *pilA* gene has been shown to impair the biofilm formation [32].

DHS01 surprisingly harboured less virulence genes than the reference strain PAO1, suggesting a lower virulence *in vivo*. This contrasts with its status of international epidemic clone. DHS01 was isolated after 25 days of patient colonization and displayed the genomic signatures of a strain adapted to chronic colonization (i.e. loss of flagellum, and deficiency in twitching motility, in the quorum sensing and in biofilm formation) [32, 34]. Although being the first isolate of the epidemic, DHS01 was likely an epidemiological dead end and was not representative of the strain that has spread to more than 300 patients [35].

### DHS01 multidrug resistance is mostly due to chromosomal mutations

DHS01 was multidrug resistant, with a high level of resistance to gentamicin (MIC >512 mg l^−1^), to tobramycin (MIC 32 mg l^−1^), to ciprofloxacin (MIC 32 mg l^−1^) and to β-lactam compounds (ticarcillin MIC 64 mg l^−1^; piperacillin MIC 128 mg l^−1^). It was of intermediate resistance to aztreonam (MIC 16 mg l^−1^), and remained susceptible to cefepime (MIC 8 mg l^−1^), ceftazidime (MIC 8 mg l^−1^), imipenem (MIC 4 mg l^−1^) and amikacin (MIC 2 mg l^−1^). The acquired genes and mutations in regulators leading to antibiotic resistance are detailed in Table 1. We had previously shown that DHS01 overproduced AmpC (650-times more than PAO1), and overexpressed *mexA* and *mexX* (1.85- and 3.70-times more than PAO1, respectively) [36]. Briefly, DHS01 produced a 2′-aminoglycoside nucleotidyl-transferase, overproduced an extended-spectrum AmpC cephalosporinase (*ampR* and *ampD* mutant), overproduced MexAB–OprM (*nalB* mutant) and MexXY (*agrZ* mutant), presumably overproduced MexEF–OprN (*mexS* mutant), and displayed canonical mutations in the QRDRs of GyrA and ParC.

**Table 1.** Acquired genes and mutations in regulators leading to antibiotic resistance in the ST395 *P. aeruginosa* isolate DHS01

**Consequence**	**Antibiotic(s) affected**	**Gene or mutation (in comparison with PAO1)**
2′-Aminoglycoside nucleotidyl-transferase production	Gentamicin, tobramycin	*ant(2′)-Ia* (borne by ICE-5)
MexXY efflux pump overproduction	Aminoglycosides, fluoroquinolones	8 nt deletion in the regulator-encoding gene *mexZ*
Quinolone target mutation	Fluoroquinolones	T83I in GyrA and S87L in ParC
AmpC cephalosporinase overproduction	Ticarcillin, piperacillin, cefepime, ceftazidime, aztreonam	M1L in the regulator AmpR, and A134V and T139M in AmpD
MexAB–OprM efflux pump overproduction	Ticarcillin, cefepime, aztreonam	H107P in the regulator MexR
MexEF–OprN efflux pump overproduction	Imipenem	R48C in the oxidoreductase MexS
Reduced production of the porin OprD	Imipenem	R48C in the oxidoreductase MexS
Production of extended-spectrum AmpC (ESAC)	Imipenem	A105 in AmpC

### ICEs in DHS01

ICEs are mobile genetic elements integrated into a genome, and able to excise and transfer by conjugation to another genome [37]. Genome analysis predicted five ICEs in the genome of DHS01 (Fig. S2). As expected, each ICE was composed of 2 to 4 GIs (Tables 2 and S4). Hence, 13 GIs were integrated into the DHS01 genome via the integration of ICEs. ICE-1, ICE-2, ICE-3, ICE-4 and ICE-5 were 78, 47, 66, 94 and 93 kb in size, respectively. None of these ICEs were retrieved in the other ST395 isolates (dataset 2) with the exception of ICE-4 shared by the strain PT F from a hospital in Birmingham, UK [5]. Fig. S2 details the structure, the gene content and the site of insertion of the five ICEs within the DHS01 chromosome. The five integrated ICEs provided DHS01 with genes involved in virulence and resistance to antibiotics and heavy metals that could have favoured its installation and spread into hospitals. Hence, ICE-4 contained the pili type IVa operon (*pilLNOXQVTM*) also present in the PAPI-1 of strain PA14 and involved in the transfer of this pathogenicity island to other strains [38]. ICE-5 harboured genes conferring resistance to aminoglycosides [*ant(2′)-Ia*], to sulfonamide (*sulI*) and to mercury (*merRTPADE*). ICE-1 and ICE-5 were integrated into the 3*′*-ends of two different tRNA^Gly^, while ICE-4 was integrated into the 3′-end of tRNA^Lys^. No tRNAs were found in the vicinity of ICE-2 and ICE-3. The activity of the ICE-4 and ICE-5 was confirmed by the existence of free circular forms after SOS stress (Fig. S3).

**Table 2.** Characteristics of the eight GIs specific of the genome of the *P. aeruginosa* ST395 isolate DHS01 These eight GIs were not retrieved in any of the 82 complete genomes of *P. aeruginosa* (Table S1). GIs in bold were specific of the clone ST395. Columns 2, 3 and 4 give the genomic coordinates (start and stop) and the length of the GI, respectively. The fifth column gives the name of the non-*P. aeruginosa* strain for which the genome contains an identical nucleotidic sequence (with >99 % length coverage and identity, unless otherwise specified). The major functions encoded by the genes harboured on the GIs are presented in the last column. The last update of the genome collection revealed that two clinical ST253 isolates (M1608 and M37351) had acquired the GI-7.

**Name**	**Start position**	**Stop position**	**Length (bp)**	**Putative species of origin**	**Encoded protein and its biological function**
GI-1	684 629	697 340	12 712	–	RNA helicase and DNA methylase (DNA processing)
GI-2	701 883	709 761	7879	–	Unknown
GI-3	2 569 006	2 615 647	46 642	–	RsmA (carbon storage regulator)
**GI-4**	**2 671 220**	**2 693 490**	22 270	***Pseudomonas putida* KT2440 (soil)**	**AlpA (regulation of prophage excision and pathogenicity)** **TetR (transcriptional regulator)** **Betaine-aldehyde dehydrogenase (choline metabolism)** **Flavin oxidoreductase (iron uptake)**
**GI-5**	**2 705853**	**2 711 674**	5822	***Acidithiobacillus ferrivorans* SS3 (96 % coverage and 82 % identity)**	**HsdS (restriction-modification)**
**GI-6**	**2 713 010**	**2723680**	10 671	–	**Heat-labile enterotoxin**
**GI-7**	**3 430 733**	**3 447 684**	16 951	***Pseudomonas putida* H8234 (93 % coverage and 99 % identity)**	**Two cupredoxins, copper ABC transporter ATPase, CopZ, CopA and CopB (copper transport)** **Helicase UvrD and endonuclease (DNA processing)**
GI-8	3 813 161	3 834 228	21 067	–	(Phage protein)

### Four GIs are ST395 specific

**Fig. 3.**
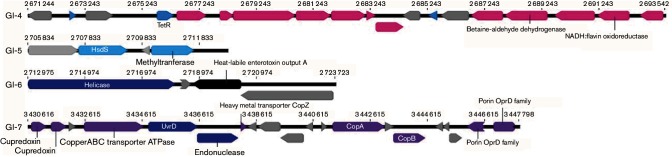
Structure of the four GIs specific to the ST395 clone. Only relevant genes are cited.

**Fig. 4.**
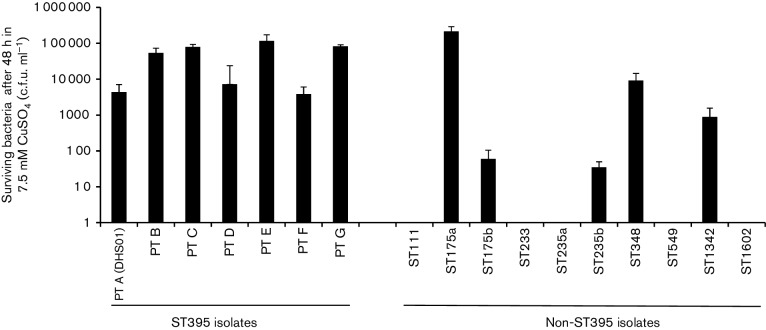
Survival of ST395 and non-ST395 isolates of *P. aeruginosa* in copper-containing media. The resistance to copper was determined in cation-adjusted MH broth supplemented with 7.5 mM CuSO_4_. Strains were described in dataset 2 (Table S2). Values are the ratio of means±sem from at least three independent experiments.

The large genome of DHS01 (7.1 Mbp) and its low G+C content suggested the presence of numerous horizontally acquired GIs. We retrieved 48 GIs in the genome of DHS01 (Table S4), among which 8 GIs were specific to DHS01 since they were not retrieved in any other strain of *P. aeruginosa* of dataset 1 (Table 2). Two of these specific GIs shared identity with sequences found in environmental proteobacteria species (*Pseudomonas putida* and *Acidithiobacillus ferrivorans*). Four of the eight DHS01-specific GIs (GI-4, GI-5, GI-6 and GI-7; Fig. 3) were also found in all the six ST395 *P* *. aeruginosa* strains tested (dataset 2), possibly identifying these four GIs as markers of the spreading capability of *P. aeruginosa* ST395 [5]. In these four ST395-specific GIs, we specifically searched for elements that could help the implantation and the spread of this international clone in hospitals. GI-4 harboured genes involved in the metabolism of choline, which is present in large amount in the outer leaflet of eukaryotic plasma membranes [39]. GI-6 harboured a gene encoding a protein that shares 58 % identity with the heat-labile enterotoxin (LT) of *Escherichia* coli for which production favours the colonization of the small intestine by enterotoxigenic *E. coli* [40] (Table 2). GI-7 contained an array of six genes encoding copper transporters: two cupredoxins, a copper ABC transporter ATPase, CopZ, CopA and CopB (Fig. 3). Cupredoxins are involved in copper homeostasis, as reported for CopC in *Pseudomonas syringae* [41]. The transporter CopZ is a copper-resistance determinant in the extremophile *Acidithiobacillus ferrooxidans* [42]. CopA and CopB are sufficient to render the cells copper resistant [43]. Hence, CopA is an inducible efflux P-type ATPase located in the inner cell membrane that exports copper from the periplasm. It also shares homology with multicopper oxidases that convert Cu^+^ into the less toxic Cu^2+^. Therefore, CopA has been thought to play a role in copper resistance through its multicopper oxidase activity and its high copper-binding capacity [44]. The gene *copB* encodes an inducible heavy metal ATPase that extrudes copper from the cell when it reaches toxic intracellular concentrations [45]. The source of the DHS01 within the University Hospital of Besançon is still under investigation; however, the role of a water network made of copper pipes has been recently suggested in a ST395 outbreak [5]. We then hypothesized that the bacterial resistance to copper helped the installation of ST395 in the hospital water network and assessed the survival of a panel of ST395 isolates in copper-containing media. All the seven ST395 tested behaved similarly, with 4.10^3^–8.10^5^ c.f.u. surviving after 48 h incubation in MH broth containing 7.5 mM CuSO_4_ (Fig. 4). This contrasted with the variations of survival of the 10 non-ST395 tested. Hence, five isolates (belonging to ST175, ST235, ST342 and ST348) could survive in copper solution, while five other isolates (belonging to ST111, ST233, ST235, ST549 and ST1602) did not. The ST395 survival in copper is presumably due to the presence of *copA* and *copB* in all ST395 isolates, but other mechanisms are implicated in copper resistance in non-ST395 strains. As expected, GI-7 containing *copA* and *copB* genes was also found in an environmental *P. aeruginosa* ST395 strain isolated from a metal-contaminated aquatic environment [7]. GI-7 was retrieved (with 99 % identity and 93 % length coverage) from a strain of *P. putida* that typically inhabits soil and water [46]. More generally, genes *copA* and *copB* are found in soil, commensal and plant pathogenic bacterial species, and may be favoured by the long-term use of sprays of copper-based bactericides on crops [47]. ST395 can contaminate hospitalized patients and those afflicted with cystic fibrosis [4, 5, 48], but is also frequently retrieved in polluted environments (estuaries, rivers, hospital wastewaters, water treatment plants) in which copper resistance could have been favoured [7, 48–51]. Our data indicate that an ST395 ancestor has horizontally acquired GI-7 from a single environmental source.

### ST395-specific CRISPR-Cas system

The CRISPR-Cas system is thought to play a role in controlling horizontal gene transfer [26]. The high number of GIs integrated into the genome of DHS01 led us to hypothesize the absence of an active CRISPR-Cas system in this epidemic strain. However, CRISPR-Finder detected two CRISPR arrays and the associated Cas proteins that correspond to the type I-E (Fig. S4). It appears to be specific to the ST395 sublineage, since it was retrieved from all the ST395 isolates tested (dataset 2) and absent from non-ST395 (datasets 1 and 2) [26]. Table S5 details the 12 and 8 spacers of the CRISPR arrays 1 and 2, respectively. Interestingly, the phage JBD25 was among the targets of one spacer of the CRISPR system, while genes of this phage were integrated into the GI-19 (Table S4). The G+C content of JBD25 genes (62.5 mol%) was much lower than that of the rest of the genome (65.8 mol%) and the prophage nucleotidic sequence was highly similar (99 % of identity) with that of the JDB25 phage. Such an integration was also found in all the other ST395, excepted in isolate PT B. This suggests a recent integration of JDB25 phage despite a dedicated CRISPR-Cas spacer, and that the system may be inactive. However, no anti-CRISPR protein I-E was retrieved in the DHS01 strain, which suggests that this system was inactivated by another mechanism, implicating possibly phage-encoded anti-CRISPR proteins [52, 53].

### Conclusion

We describe here the large genome (7.1 Mbp) of the first isolate of the international clone of *P. aeruginosa* ST395 that further spread into our university hospital during at least 11 years. The majority of the 1555 accessory genes were brought by the many GIs (*n*=48) and ICEs (*n*=5) integrated into the DHS01 chromosome. The multidrug resistance of the isolate was mostly due to mutations in antibiotic targets, and in regulators which mutations trigger the overproduction of cephalosporinase and efflux pumps. DHS01 was retrieved from a chronically-colonized patient and displayed the genomic signatures of adaptation to chronic infection mostly due to the deletion of a 131 kbp chromosomal fragment. Four GIs were seemingly specific to the widespread clone ST395. They harboured genes involved in metabolism, in virulence and in the resistance to copper, which was confirmed phenotypically in a panel of independent ST395 isolates. Resistance to copper possibly accounts for the spreading capability of ST395 in hospital settings in which the water network may be incriminated [5]. This suggests that genes selected in environmental bacterial species by the use of heavy metals can be transferred to pathogens that further spread into hospital settings.

## Data bibliography

1. Valot B, Rohmer L, Jacobs MA, Miller SI, Bertrand X *et al*. NCBI accession number CP013993.1 (2016).
2. Rogues AM, Cholley P, Petitjean M, Bertrand X, Hocquet D. NCBI BioProject PRJNA379554 (2017)
3. Bertrand X, Sauget M, Petitjean M, Hocquet D. NCBI BioProject PRJNA380885 (2017).
4. Multiple authors, sees Table S1 and S2. (June 2017).

## Supplementary Data

870 Supplementary File 1
