# Supplementary material for: Genomic characterization of a local epidemic Pseudomonas aeruginosa reveals specific features of the widespread clone ST395
Source: Microb Genom. 2017 Jun 8;3(10):e000129. doi: 10.1099/mgen.0.000129 (PMC5695207; doi:10.1099/mgen.0.000129)

Table S1: Characteristics of the 84 complete genomes of *P. aeruginosa* extracted from NCBI, dataset 1 (June 2017).

Strain	Origin	Length (bp)	Number of genes	ST	NCBI accession number
Carb01-63	Clinical (unknown)	7497593	6910	111	NZ_CP011317.1
RIVM-EMC2982	Clinical (unknown)	7380063	6916	111	NZ_CP016955.1
F30658	Clinical (unknown)	7273258	6717	111	NZ_CP008857.1
NCGM257	Clinical (urine)	7090694	6712	357	NZ_AP014651.1
E6130952	Clinical (sputum)	7040952	6631	235	NZ_CP02603.1
PA7790	Clinical (tracheal aspirate)	7018690	6645	277	NZ_CP014999.1
PA11803	Clinical (bloodstream)	7006578	6637	277	NZ_CP015003.1
PASGNDM699	Clinical (sputum)	6985102	6545	308	NZ_CP020704.1
S86968	Clinical (unknown)	6934277	6399	155	NZ_CP008865.2
PA8281	Clinical (tracheal aspirate)	6928736	6461	277	NZ_CP015002.1
M37351	Clinical (unknown)	6897231	6387	253	NZ_CP008863.1
IOMTU-133	Clinical (urinary catheter)	6897018	6338	1047	NZ_AP017302.1
W60856	Clinical (unknown)	6896700	6376	959	NZ_CP008864.2
PASGNDM345	Clinical (sputum)	6893164	6432	308	NZ_CP020703.1
FA-HZ1	Environmental (wastewater)	6866790	6209	27	NZ_CP017353.1
NCGM-1984	Clinical (urinary catheter)	6850954	6257	235	NZ_AP014646.1
H47921	Clinical (unknown)	6836415	6306	1105	NZ_CP008861.1
ATCC-27853	Clinical (unknown)	6827737	6376	155	NZ_CP015117.1
VRFPA04	Clinical (eye)	6818030	5841	823	NZ_CP008739.1
NCGM-1900	Clinical (urinary catheter)	6814936	6206	235	NZ_AP014622.1
W16407	Clinical (unknown)	6808844	6234	244	NZ_CP008869.2
T38079	Clinical (unknown)	6795741	6176	155	NZ_CP008866.2
F9670	Clinical (unknown)	6794354	6028	155	NZ_CP008873.1
W36662	Clinical (unknown)	6791731	6286	17	NZ_CP008870.2
W45909	Clinical (unknown)	6777566	6299	27	NZ_CP008871.2
NCGM2-S1	Clinical (urine)	6764661	6140	235	NC_017549.1/
SCV20265	Clinical (cystic fibrosis sputum)	6725183	6131	299	NC_023149.1/
B10W	Environmental (wastewater)	6723378	6216	308	NZ_CP017969.1
PA1088	Clinical (urine)	6721480	6247	277	NZ_CP015001.1
FRD1	Clinical (cystic fibrosis sputum)	6712339	5871	111	NZ_CP010555.1
PA-D25	Clinical (sputum)	6682304	6143	1971	NZ_CP012584.1
PA-D5	Clinical (sputum)	6681992	6153	1971	NZ_CP012579.1
PA-D22	Clinical (sputum)	6681981	6146	1971	NZ_CP012583.1
PA-D16	Clinical (sputum)	6681975	6150	1971	NZ_CP012581.1
PA-D9	Clinical (sputum)	6645477	6119	1971	NZ_CP012580.1
PA-D1	Clinical (sputum)	6643823	6120	1971	NZ_CP012585.1
PA-D2	Clinical (sputum)	6642996	6120	1971	NZ_CP012578.1
DN1	Environmental (soil)	6641902	6535	316	NZ_CP017099.1
PA-D21	Clinical (sputum)	6639108	6116	1971	NZ_CP012582.1
F63912	Clinical (unknown)	6618768	6186	198	NZ_CP008858.2

8380	Clinical (gut)	6613260	6150	Unknown	NZ_AP014839.1
F22031	Clinical (unknown)	6603099	5968	485	NZ_CP007399.1
LESB58	Clinical (cystic fibrosis sputum)	6601757	6005	146	NC_011770.1
PA7	Clinical (unknown)	6588339	5932	1195	NC_009656.1
MTB-1	Environmental	6580038	5953	Unknown	NC_023019.1
H27930	Clinical (unknown)	6568228	5982	389	NZ_CP008860.1
LES431	Clinical (unknown)	6550070	5925	146	NC_023066.1
PA14OR	Unknown	6541482	6038	253	NZ_LT608330.1
PA14	Clinical (burn)	6537648	5873	253	NC_008463.1
F23197	Clinical (unknown)	6517340	5941	1295	NZ_CP008856.2
PAER4-119	Unknown	6504659	5984	260	NZ_CP013113.1
PA1RG	Clinical (unknown)	6500439	5917	782	NZ_CP012679.1
PA1	Clinical (respiratory tract infection)	6498072	5910	782	NC_022808.2
M1608	Clinical (unknown)	6460023	5934	253	NZ_CP008862.2
T63266	Clinical (unknown)	6456866	5925	132	NZ_CP008868.1
PA-154197	Unknown	6445239	5961	550	NZ_CP014866.1
YL84	Environmental	6433441	5785	(CC169)	NZ_CP007147.1
12-4-4-59	Clinical (blood culture of burn patient)	6431911	5912	152	NZ_CP013696.1
PA121617	Clinical (sputum)	6430493	6367	389	NZ_CP016214.1
B136-33	Clinical (unknown)	6421010	5771	1024	NC_020912.1
DK2	Clinical (cystic fibrosis sputum)	6402658	5788	386	NC_018080.1
VA-134	Clinical (skin wound of burn patient)	6400418	5850	Unknown	NZ_CP013245.1
N17-1	Environmental (soil)	6370730	5899	2362	NZ_CP014948.1
F9676	Environmental	6368008	5721	167	NZ_CP012066.1
USDA-ARS-USMARC-41639	Nasopharyngeal swab of Bos Taurus	6364583	5856	Unknown	NZ_CP013989.1
X78812	Clinical (unknown)	6348761	5807	257	NZ_CP008872.2
AES-1R	Clinical (cystic fibrosis sputum)	6343337	5888	649	NZ_CP013680.1
RP73	Clinical (cystic fibrosis sputum)	6342034	5724	198	NC_021577.1
H5708	Clinical (unknown)	6334378	5829	Unknown	NZ_CP008859.2
M18	Environmental	6327754	5717	1239	NC_017548.1
T52373	Clinical (unknown)	6322459	5691	Unknown	NZ_CP008867.1
DSM-50071	Clinical (unknown)	6317050	5709	Unknown	NZ_CP012001.1
NCTC10332	Unknown	6316979	5761	Unknown	NZ_LN831024.1
PA1R	Clinical (unknown)	6309305	5752	782	NC_022806.1
PcyII-10	Unknown	6288645	5770	1233	NZ_LT673656.1
PAO1OR	Orsay varient of PAO1 strain	6276469	5797	549	NZ_LN871187.1
ATCC-15692	Clinical (wound)	6276434	5782	549	NZ_CP017149.1
PAO1	Clinical (wound)	6264404	5572	549	NC_002516.2
SJTD-1	Environmental (soil)	6243825	5739	Unknown	NZ_CP015877.1
Nhmuc	Unknown	6213276	5708	387	NZ_CP013479.1
SCVJan	Murine model	6213029	5704	387	NZ_CP013478.1
SCVFeb	Murine model	6213026	5704	387	NZ_CP013477.1

Strain	Origin	Availability	ST	Reference
PT B	Hospital of Besançon, France	BioSample SAMN06627436	395	This study
PT C	Hospital of Besançon, France	BioSample SAMN06627437	395	This study
PT D	Hospital of Lyon, France	BioSample SAMN06627442	395	This study
PT E	Hospital of Lyon, France	BioSample SAMN06627446	395	This study
PT F (Birmingham 903)	Hospital of Birmingham, UK	European Nucleotide Archive ERP006058	395	Quick J *et al.* BMJ Open. 2014 Nov 4;4(11):e006278.
PT G	Hospital of Bordeaux, France	BioSample SAMN06627443	395	This study
ST111	Hospital of Besançon, France	BioSample SAMN06651179	111	This study
ST175a	Hospital of Besançon, France	BioSample SAMN06627435	175	This study
ST175b	Hospital of Besançon, France	BioSample SAMN06651181	175	This study
ST233	Hospital of Besançon, France	BioSample SAMN06651183	233	This study
ST235a	Hospital of Besançon, France	BioSample SAMN06651178	235	This study
ST235b	Hospital of Besançon, France	BioSample SAMN06651180	235	This study
ST348	Hospital of Nancy, France	BioSample SAMN06651182	348	This study
PAO1	Melbourne, Australia	NCBI Reference Sequence: NC_002516.2	549	Stover CK *et al.* Nature. 2000 Aug 31;406(6799):959-64.
ST1342	Hospital of Lariboisière, Paris, France	BioSample SAMN06627441	1342	This study
ST1602	Hospital of Lyon, France	BioSample SAMN06627438	1602	This study

Table S2: Informations about raw data of *P. aeruginosa* ST395 and non-ST395 strains used for characterisation of specific genetic material, dataset 2.

ICE number	PCR	Forward primer sequence (5'-3')	Reverse primer sequence (5'-3')
ICE-1	Initial	GGATCTGCAAGGCCAGGTTG	GCAGGCTGAGAGCAGGATAG
	Nested	GCGAACTGATCGCCGGG	TCGTTGTCGGAGAGGGTGTA
ICE-2	Initial	GTGGATGGGGATCTACTGCC	TGAGCTGGTTGTTCTGGTCG
	Nested	CATTGCTACGCCTGCAGAAT	GCAAAATGCACCGAGGTTGG
ICE-3	Initial	TGACTTGATCGGCGTAACCC	CGCATGTATCTTCCCCGGTT
	Nested	TGTACGCCCTTCTTGACGAG	ATTTCCCCGCCATAGGTAGC
ICE-4	Initial	CCTGTTCTTCGCTGGATCGT	ATGACGGGCATCTTTCAGGG
	Nested	CTCCCTTCCTGATCGCAGTG	AGGTCTGAAAGCATGAGCCC
ICE-5	Initial	GATACTGAAGGGCCGGCTAC	GAAGTGAAGTTCCTCGGCCA
	Nested	CGCCTGCAGAATGGTTTCAG	CCGGTTCCTGGTAACGTCTG

Table S3: Primers used for the identification of circular forms of the ICEs using nested PCR.

Name	Start	Stop	Length (bp)	Geno-me(s) (*n*)	Putative species of origin	Function
GI-9	676826	683715	6890	65	-	(Phage protein)
GI-10	775197	816878	41682	84	-	DNA processing, defense mechanisms & secondary metabolites biosynthesis, transport and catabolism.
GI-11	676,826	683,715	6,890	10	-	(Phage protein)
GI-12	775,197	816,878	41,682	16	-	DNA processing, defense mechanisms & secondary metabolites biosynthesis, transport and catabolism.
GI-13	909,813	914,334	4,522	1	-	Unknown
GI-14	914,744	948,980	34,237	4	-	(Phage protein & regulation)
GI-15	1,112,226	1,117,419	5,194	2	*Pseudomonas* phage MP22 / Phage F_HA0480sp/Pa1651	(Phage protein)
GI-16	1,117,436	1,123,713	6,278	1	*Pseudomonas* phage JBD93	(Phage protein)
GI-17	1,171,634	1,186,830	15,197	11	-	DNA processing
GI-18	1,190,408	1,198,422	8,015	4	*Pseudomonas fluorescens / Pseudomonas balearica*	DNA processing
GI-19	1,363,454	1,379,352	15,899	3	*Pseudomonas* phage JBD25	(Phage protein)
GI-20	1,395,087	1,425,084	29,998	4	-	(Phage protein)
GI-21	2,151,362	2,169,893	18,532	15	-	Sugar metabolism & cell wall/membrane/ envelope biogenesis
GI-22	2,551,027	2,557,727	6,701	1	-	Unknown

GI-23	2,793,073	2,797,178	4,106	26	-	Intracellular trafficking, secretion and vesicular transport
GI-24	2,893,400	2,903,896	10,497	5	*Pseudomonas misselii*	Metabolism (ICE-1)
GI-25	2,966,826	2,974,108	7,283	18	-	Metabolism (ICE-1)
GI-26	3,420,507	3,429,999	9,493	6	-	DNA processing
GI-27	3,451,116	3,466,146	15,031	9	-	DNA processing & lipid and copper transport
GI-28	3,472,217	3,479,103	6,887	20	-	Metabolism
GI-29	3,620,322	3,628,120	7,799	8	-	DNA processing & amino acid transport and metabolism
GI-30	3,789,125	3,798,230	9,106	2	-	Unknown
GI-31	3,860,394	3,864,454	4,061	6	-	Unknown
GI-32	4,033,634	4,087,975	54,342	2	-	(Phage protein)
GI-33	4,326,868	4,331,361	4,494	7	*K. pneumoniae* plasmid / *E. cloacae* plasmid	Conjugation machinery (ICE-2)
GI-34	4,331,871	4,344,728	12,858	3	*K. pneumoniae* plasmid / *E. cloacae* plasmid	Resistance and virulence (ICE-2)
GI-35	4,358,031	4,362,326	4,296	5	*K. pneumoniae* plasmid / *E. cloacae* plasmid	DNA processing (ICE-2)
GI-36	4,713,679	4,717,717	4,039	11	-	Virulence (Pyocin S2)
GI-37	4,782,199	4,789,393	7,195	6	-	Metabolism
GI-38	4,871,972	4,891,972	20,001	2	-	Unknown
GI-39	4,893,999	4,913,052	19,054	1	-	(Phage protein)
GI-40	5,004,422	5,008,882	4,461	23	-	Metabolism & repair system
GI-41	5,010,115	5,015,280	5,166	11	-	DNA processing (ICE-4)

GI-42	5,020,832	5,027,255	6,424	13	-	Regulation & DNA processing (ICE-4)
GI-43	5,055,166	5,062,300	7,135	5	*Pseudomonas aeruginosa* genomic island PAGI-5	Unknown (ICE-4)
GI-44	5,365,376	5,375,164	9,789	9	*Klebsiella oxytoca*	Unknown (ICE-4)
GI-45	5,411,217	5,440,266	29,050	4	-	Resistance & DNA processing (ICE-5)
GI-46	5,443,133	5,459,224	16,092	1	-	Regulation & DNA processing (ICE-5)
GI-47	5,467,263	5,473,009	5,747	1	-	Unknown
GI-48	6,538,381	6,550,792	12,412	6	-	DNA processing

Table S4: Characteristics of the 40 genomic islands (GIs) non-specific of the genome of the *P. aeruginosa* ST395 isolate DHS01.

CRISPR array	Spacer number	Phage name	GenBank accession number
1	3	*Pseudomonas* phage vB_PaeP_Tr60_Ab31	HG798806.1
	5	Bacteriophage D3112	AY394005.1
		Pseudomonas phage H70	M233689.1
		Pseudomonas phage PaMx73	JQ067085.2
		Pseudomonas phage JBD24	JX434031.1
		Pseudomonas phage JBD25	JX495042.1
		Pseudomonas phage JBD26	JN811560.1
		Pseudomonas phage DMS3	DQ631426.1
		Bacteriophage D3112	AY394005.1
	6	*Pseudomonas* phage DMS3	DQ631426.1
		Pseudomonas phage vB_PaeS_PAO1_Ab30	LN610590.1
		Pseudomonas phage JBD24	JX434031.1
		Pseudomonas phage MP38	EU272037.1
	8	*Pseudomonas* phage DO4	KM389210.1
	10	Bacteriophage F10	DQ163912.1
		Pseudomonas phage phi2	KT887558.1
2	2	Bacteriophage D3112	AY394005.1

Table S5: Correlation between spacers in CRISPR and bacteriophages

Fig. S1: Pulsed-field gel electrophoresis profiles of *Dra*I-digested DNA from *P. aeruginosa* ST395 isolates (n=7).

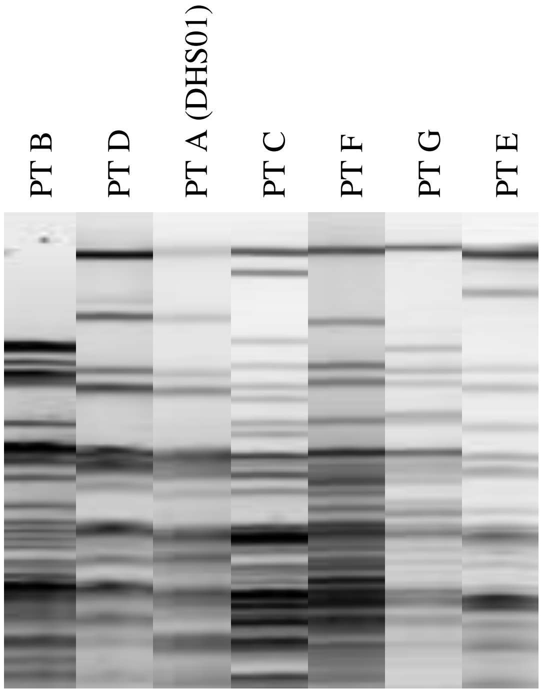

Fig. S2: Composition of the five ICEs of the isolate DHS01.

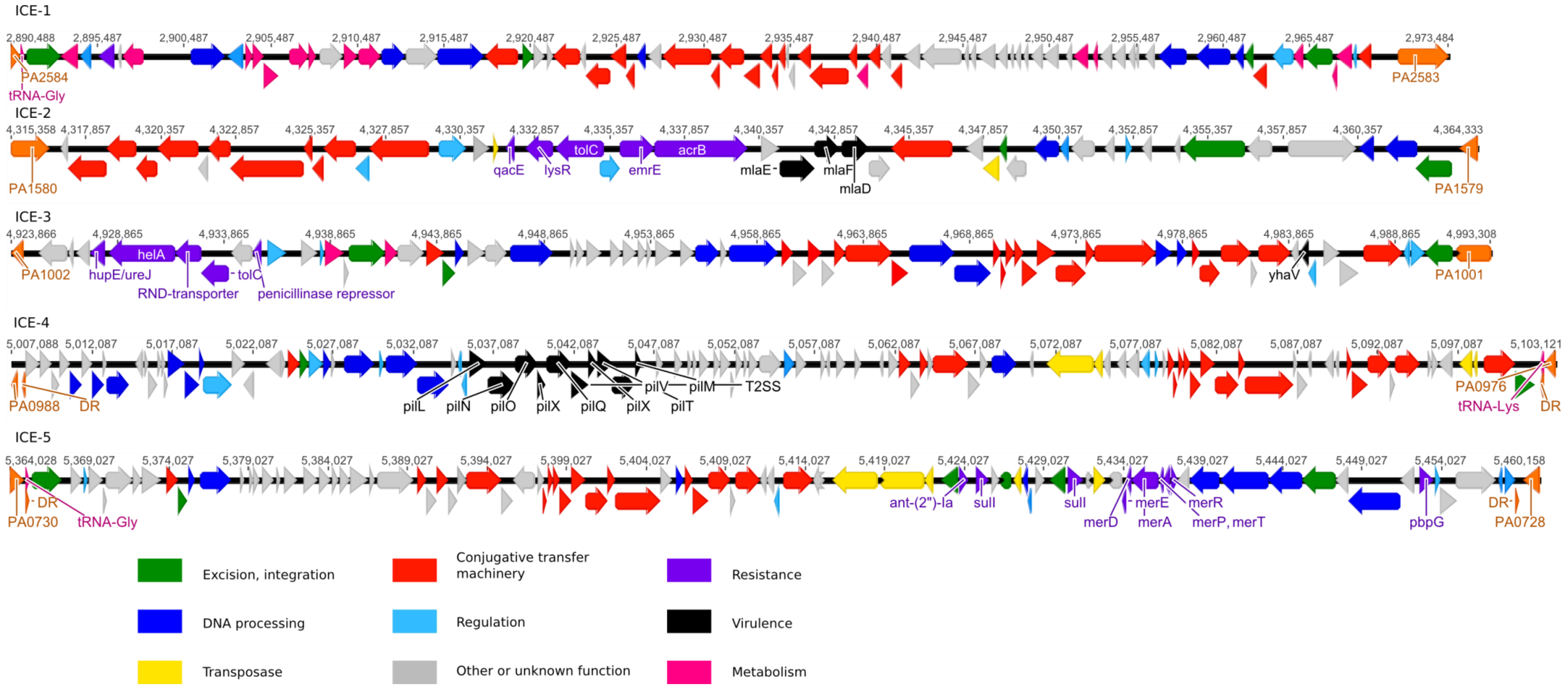

Fig. S3: Agarose gel analyses of fragments of circular ICE form after PCR..

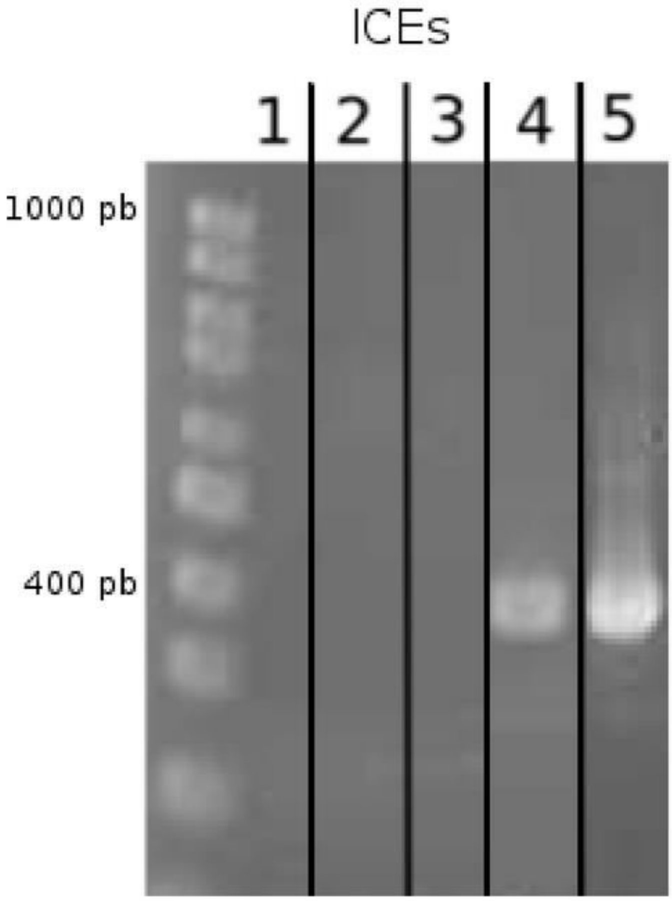

Fig. S4: Structure of the two type I-E CRISPR arrays and Cas proteins associated in the genomes of *Pseudomonas aeruginosa* ST395 isolates.

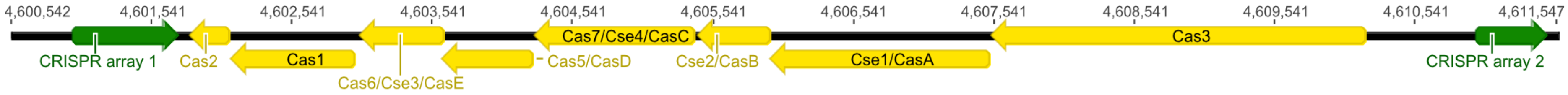

Supplement: Supplementary File 1 [file mgen-3-129-s001.pdf]
